# Linking specific biological signatures to different childhood adversities: findings from the HERO project

**DOI:** 10.1038/s41390-022-02415-y

**Published:** 2023-01-17

**Authors:** Euclides José de Mendonça Filho, Irina Pokhvisneva, Christina Maria Maalouf, Carine Parent, Shanna B. Mliner, Natalie Slopen, David R. Williams, Nicole R. Bush, William Thomas Boyce, Pat Levitt, Charles A. Nelson, Megan R. Gunnar, Michael J. Meaney, Jack P. Shonkoff, Patricia Pelufo Silveira, on behalf of the JPB Research Network on Toxic Stress

**Affiliations:** 1 Ludmer Centre for Neuroinformatics and Mental Health, Douglas Hospital Research Center, Montreal, QC Canada; 2 Department of Psychiatry, McGill University, Montreal, QC Canada; 3 Institute of Child Development, University of Minnesota, Minneapolis, MN USA; 4 Department of Social and Behavioral Sciences, Harvard T.H. Chan School of Public Health, Harvard University, Boston, MA USA; 5 Department of Psychiatry and Behavioral Sciences, Weill Institute for Neurosciences, San Francisco, CA USA; 6 Division of Developmental Medicine, Department of Pediatrics, University of California, San Francisco, San Francisco, CA USA; 7 Department of Pediatrics and Program in Developmental Neuroscience and Developmental Neurogenetics, The Saban Research Institute, Children’s Hospital Los Angeles, Keck School of Medicine, University of Southern California, Los Angeles, CA USA; 8 Boston Children’s Hospital and Harvard Medical School, Boston, MA USA; 9 Harvard Graduate School of Education, Cambridge, MA USA; 10 Singapore Institute for Clinical Sciences, Agency for Science, Technology and Research (A*STAR), Brenner Centre for Molecular Medicine, Singapore, Republic of Singapore; 11 Center on the Developing Child, Harvard University, Cambridge, MA USA

## Abstract

**Background:**

Although investigations have begun to differentiate biological and neurobiological responses to a variety of adversities, studies considering both endocrine and immune function in the same datasets are limited.

**Methods:**

Associations between proximal (family functioning, caregiver depression, and anxiety) and distal (SES-D; socioeconomic disadvantage) early-life adversities with salivary inflammatory biomarkers (IL-1β, IL-6, IL-8, and TNF-α) and hair HPA markers (cortisol, cortisone, and dehydroepiandrosterone) were examined in two samples of young U.S. children (*N* = 142; *N* = 145).

**Results:**

Children exposed to higher SES-D had higher levels of TNF-α (*B* = 0.13, *p* = 0.011), IL-1β (*B* = 0.10, *p* = 0.033), and DHEA (*B* = 0.16, *p* = 0.011). Higher family dysfunction was associated with higher cortisol (*B* = 0.08, *p* = 0.033) and cortisone (*B* = 0.05, *p* = 0.003). An interaction between SES-D and family dysfunction was observed for cortisol levels (*p* = 0.020) whereby children exposed to lower/average levels of SES-D exhibited a positive association between family dysfunction and cortisol levels, whereas children exposed to high levels of SES-D did not. These findings were partially replicated in the second sample.

**Conclusions:**

Our results indicate that these biological response systems may react differently to different forms of early-life adversity.

**Impact:**

- Different forms of early-life adversity have varied stress signatures, and investigations of early-life adversities with inflammation and HPA markers are lacking.
- Children with higher socioeconomic disadvantage had higher TNF-α, IL-1β, and DHEA.
- Higher family dysfunction was associated with higher hair cortisol and cortisone levels, and the association between family dysfunction and cortisol was moderated by socioeconomic disadvantage.
- Biological response systems (immune and endocrine) were differentially associated with distinct forms of early-life adversities.

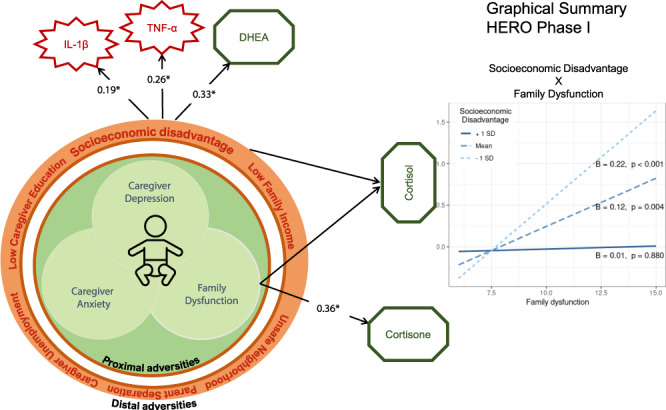

## Introduction

Early-life adversity can be conceptualized as a violation of the expectable developmental environment by exposure to harmful or threatening stimuli (e.g., biological or psychosocial hazards), absence of responsive caregiving needed for healthy development, or both.^1,2^ Exposure to early-life adversities increases the risk of developing psychological and physical health problems well into the adult years.^3,4^ The long-term consequences of early adversity are substantial as these individuals account for 29.8% of all psychiatric cases across 21 countries.^5^ Individuals exposed to adversity early in life also have a 44% greater likelihood of heart attacks and strokes,^6^ and an increased risk of all-cause mortality.^7^ Likewise, these individuals also have a greater risk for auto-immune disorders such as asthma, with varying risk intensity depending on the type of adversity experienced.^8^ Despite substantial evidence for adversity–health associations, the underlying mechanisms involved in the developmental pathway from early-life adversity to future pathology are not well understood and the early identification of the effects of different forms of adversity remains a challenge.^3^

One of the reasons for this knowledge gap is the fact that more research have been conducted on the effects of adversity on school-age children, adolescents, and adults, with little known about effects in early childhood.^9^ The definition of early adversity is another aspect that is often left imprecise due to its different domains and different levels. Nevertheless, a notable feature is that such exposures require different adaptive responses by the child.^10^ Psychosocial adversity includes several domains that can be generally disentangled into two forms. One form comprises proximal adverse experiences that impact children directly, which includes various forms of abuse or neglect, family psychiatric problems (e.g., maternal mental health), disruptions in family functioning, witnessing domestic violence, loss of a caregiver, and others.^11^ Another form of adversity encompasses conditions that affect the child indirectly, here defined as broader contextual/distal adversities.^9^ These include conditions such as growing up in poverty, stresses on families associated with systemic racism and interpersonal discrimination, living in a violent neighborhood, and occupational or academic disruption in the lives of caregivers.^10–12^ Although not as apparent as being the target of maltreatment, distal adversities are thought to affect children indirectly through a cascade of events. These influences include distress in primary caregivers which can undermine the quality of caregiver–child interactions that moderate negative emotions and physiological processes associated with noxious stimulation.^9,13^

Different forms of early adversity may have varied stress signatures and their impact on child’s development might occur through differential effects on distinct biological systems.^6,8,14,15^ One biological mechanism commonly associated with poor child outcomes and future pathology is disrupted reactivity and regulation of stress responses by the hypothalamic–pituitary–adrenocortical (HPA) axis.^16^ Activation of the HPA axis in response to stress results in the release of cortisol to mobilize energy and resources to respond to environmental threats.^17^ This mobilization allows the organism to maintain functioning under challenging conditions (allostasis), but under conditions of chronic stress (high allostatic load), in a sensitive period of development, the organism might exhibit a maladaptive HPA response.^18^ Along with cortisol, dehydroepiandrosterone (DHEA) and its sulfate ester (DHEAS) are also secreted in response to stress.^19^ More recently, attention has shifted to the interplay between DHEA and cortisol as several physiological systems are affected by both hormones. For example, DHEA can antagonize effects of cortisol actions and biological actions of DHEA also involve anti-inflammatory, neuroprotective, and antioxidant effects.^19^ Thus, taking cortisol and DHEA hormones with the inactive metabolite of cortisol (i.e., cortisone) into account may be a more sensitive indicator of HPA axis activity.^20^ Variation in HPA axis function due to stress has been shown to have a significant impact on neural development and behavior in animal models.^21^ In humans, the effects of prolonged early-life deprivation can also result in future disrupted HPA axis response to stress.^22–24^ This suggests that timing is particularly relevant since maladaptive responses during a sensitive developmental period can lead to abiding structural alterations and physiologic dysregulations.^1,4^

Another pathway through which early adversity appears to influence mental and physical health is via modification of the immune system through an imbalance of inflammatory processes.^25,26^ Inflammation is the body’s initial defense against viral and bacterial infection. However, chronic inflammation is associated with the pathogenesis of depression and cardiovascular disease, and higher levels of inflammatory markers have been implicated in poorer neurodevelopment in children growing up in adverse environments.^3,6,27^

The impact of different forms of adversity on both the immune system and the HPA axis, and the bidirectional communication between these systems is still unclear. For example, the cytokines tumor necrosis factor-α (TNF-α), interleukin-1 (IL-1), and interleukin-6 (IL-6) act as a stimulus for HPA axis activity in plasma, while cortisol suppresses proinflammatory and activates anti-inflammatory responses.^28,29^ Thus, in the context of chronic early-life adversity, where blunted HPA axis activity is commonly observed, diminished HPA axis activity may contribute to the dysregulation of the immune system, reducing the inhibition of inflammatory processes. This bidirectional relation emphasizes the relevance of considering how alterations in these two systems influence each other, and potentially the co-regulation of other biological systems, that together underlie mental and physical pathogenesis.^10,28,29^

To our knowledge, there is a paucity of studies that have examined the associations of different forms of early adversity and accompanying stress signatures on both the immune and HPA axis systems specifically during the first 5 years of life. A recent study that considered both proximal and contextual adversities only evaluated the HPA axis response in infants.^16^ Most studies of inflammatory biomarkers have been conducted in premature infants^30^ and children with autism spectrum disorder,^31^ but have not investigated differences in proximal versus contextual types of early-life adversities.^26,27^ Recent investigations have started to differentiate distinct forms of adversity in which contextual stressors, parental stress, the number of traumatic events to which children were exposed, and attachment styles predicted salivary inflammatory responses,^32,33^ but studies considering both biological systems together are still rare. In order to investigate how different forms of psychosocial adversity impact those two biological systems, our current study aimed to test the associations of proximal (e.g., family functioning, caregiver’s depression, and anxiety) and distal (e.g., socioeconomic disadvantage) early-life adversities with salivary inflammatory biomarkers (IL-6, IL-8, TNF-α, and IL-1β cytokines) and a panel of hair HPA axis markers (cortisol, cortisone, and DHEA) in young children between 3 months and 5 years of age. This study is part of the Health’s Early Roots & Origins (HERO) project, a project constituted of two independent samples (HERO Phase-I and HERO Phase-II). We also investigated the effect of interaction between socioeconomic disadvantage (distal adversity) and family dysfunction (proximal adversity) based on previous interactions reported by Johnson et al.^16^

## Methods

### Participants

We interrogated data from the Phase-I and Phase-II feasibility stages of the HERO project. HERO is a unique initiative resulting from the combined efforts of scientists, pediatric practitioners, and community leaders that compose the JPB Research Network on Toxic Stress, driving a transformative agenda to establish and validate biological measures of stress activation in healthy children at scheduled well-child visits between 4 months and 5 years of age.

Phase-I was defined as a discovery sample in the current study. It comprised a sample of 142 caregivers (87.3% mothers; 12.7% fathers) and children with ages 3–68 months (MD = 18, IQR = 27) recruited from four affiliated pediatric clinics in Austin, TX; Bronx, NY; Cambridge, MA; and Albuquerque, NM from November 2017 to September 2018. Caregivers were American-born (48.2%) and answered the questionnaires according to their language preference. Phase-II was set as a replication sample in the current study. Its participants were 145 young children and their caregivers (52.4% American born, 87.5% mothers; 11.8% fathers; and 0.7% grandmothers). In the second sample children’s age was significantly different from Phase-I and ranged from 3 to 61 months with the mean age around 12 months (MD = 12, IQR = 10). For the second phase, data collection took place in the same pediatric clinics from December 2018 to September 2019. We also observed significant differences in family household income in which Phase-2 families had higher annual income, the demographics of both samples are shown in Table 1.

**Table 1:** Study sample characteristics.

Variable	Phase-I	Phase-II	*p*
	Summary statistic	Summary statistic	
Child age in months, M (SD)	24.96 (18.32)	12.83 (8.51)	*p* < 0.001
Child sex			
Male	74 (52.1%)	70 (48.3%)	0.578
Female	68 (47.9%)	75 (51.7%)	
Ethnicity			
Hispanic	76 (53.9%)	45 (31.7%)	0.728
White	42 (29.8%)	69 (48.6%)	
Black	13 (9.2%)	18 (12.7%)	
Other (Asian, Native Hawaiian, or Pacific Islander)	10 (7.1%)	10 (7.0%)	
Family household income			
<$15,000 per year	22 (15.5%)	3 (3.1%)	*p* < 0.001
$15,001–$30,000 per year	24 (16.9%)	38 (39.2%)	
$30,001–$60,000 per year	30 (21.1%)	12 (12.4%)	
$60,001–$90,000 per year	11 (7.7%)	11 (11.3%)	
≥$90,001 per year	42 (29.6%)	33 (34.0%)	
Maternal education			
Some high school or less	29 (20.4%)	27 (19.4%)	0.592
General education diploma or High school diploma	29 (20.4%)	31 (22.3%)	
Some college or complete community college	28 (19.0%)	25 (18.0%)	
Four-year degree or some graduate school	26 (18.3%)	17 (12.2%)	
Master’s degree or higher	26 (18.3%)	39 (28.1%)	

### Procedures

Biological samples were collected from children, and psychosocial data were informed by caregivers in the context of well-child pediatric visits. Samples were collected through non-invasive methods which included cheek swabs for buccal epithelial cells, a small amount of saliva to measure inflammatory markers such as cytokines, and several strands of hair to measure steroid levels. Children’s anthropometric measurements were also obtained, including height, weight, and head circumference. Children with pre-term birth, congenital disorders, previous recent vaccination, and steroid medication use were not eligible to participate in the study. The surveys and data management protocols were set up using the Research Electronic Data Capture (REDCap) tools. All participants provided informed written consent, and study procedures were approved by the local institutional review board for each pediatric clinic involved in the HERO project (Young Children’s Health Center, Albuquerque, NM; People’s Community Clinic, Austin, TX; Yogman Pediatric, Cambridge, MA; Albert Einstein College of Medicine, Bronx, NY) and the Douglas Research Centre (Research Ethics Board – Mental Health and Neuroscience subcommittee affiliated with McGill University and the Montreal West Island IUHSSC).

### Biochemical measures

#### Pro-inflammatory markers

Samples were obtained from children during a single visit to a HERO-affiliated pediatric clinic using SalivaBio Infant’s Swabs for infants aged 0.15–6 months, and SalivaBio ORAL Swabs for participants aged >6 months. If children drank milk/formula or had eaten any food within 45 min before sample collection, parents were asked to give bottled water to rinse their mouths and then wait 15 min before sampling. Saliva was stored at −20 °C and shipped to the Douglas Mental Health University Institute (Montreal, QC, Canada) where it was aliquoted and forwarded to be processed at the Salimetrics Lab and Technology Center (Carlsbad, CA) for assay. IL-1β, IL-6, IL-8, and TNF-α cytokines were measured in duplicate using a multiplex assay (one sample, multiple data points per well on the plate) by enzyme-linked immunosorbent assay (ELISA), using a proprietary method developed and validated for saliva by the Salimetrics Lab and Technology Center. As cytokines were measured in duplicate, their values were averaged. In Phase-I, the minimum detectable concentrations of IL-1β, TNF-α, IL-6, and IL-8 were 1.59, 0.01, 0.29, and 19.53 pg/mL, respectively. The average assay range was 600 pg/mL for IL-1β, 20 pg/mL for TNF-α, 105 pg/mL for IL-6, and 4919 pg/mL for IL-8. The intra-individual coefficient of variation was between 0 and 12% for IL-1β, 0 and 104.3% for TNF-α, 0 and 20.6% for IL-6, and 0 to 59.9% for IL-8. We considered the criteria used by McKay et al.^34^ for intra-individual variation reliability, and therefore cytokines’ measurements were excluded from the analyses if the coefficient of variation was higher than 15%. This led to the exclusion of 3, 4, and 69 samples for IL-8, IL-6, and TNF-α, respectively. In Phase-II, the minimum detectable concentrations of IL-1β, TNF-α, IL-6, and IL-8 were 0.31, 0.19, 0.16, and 0.07 pg/mL, respectively. The average assay range was 292 pg/mL for IL-1β, 14.2 pg/mL for TNF-α, 110 pg/mL for IL-6, and 4340 pg/mL for IL-8. The intra-individual coefficient of variation ranged from 0 and 0.1% for IL-1β, 0 and 0.3% for TNF-α, 0 and 0.3% for IL-6, and 0 to 0.2% for IL-8.

#### HPA axis markers

A steroid panel of cortisol, cortisone, testosterone, progesterone, and DHEA was obtained from the hair of a sub-sample of children (Phase-I: *N* = 79, Phase-II: *N* = 54), subject to sufficient hair to cut and parent consent. Hair was cut as close to the scalp as possible, at approximately 2 cm below the cranial bone, and the scalp-end of hair strands were marked. Samples were then shipped to be analyzed at the Dresden LabService (Technische Universität Dresden, Germany). Hair samples with lengths varying from 1 to 3 cm were weighted and washed in 2.5 mL isopropanol for 3 min at room temperature. After extraction, samples were quantified by high-performance liquid chromatography–mass spectrometry.^35^ We did not observe a significant association between hair mass and cortisol, cortisone, and DHEA concentrations (see Supplementary Table S1). The minimum detectable concentrations of cortisol, cortisone, DHEA, progesterone, and testosterone were 0.53, 0.48, 1.35, 0.97, and 0.52 pg/mg respectively in Phase-I. Three children had non-detectable levels of DHEA, and 55 and 22 presented non-detectable levels of testosterone and progesterone, respectively. In Phase-II, the minimum detectable concentrations of cortisol, cortisone, and DHEA were 0.89, 4.89, and 0.96 pg/mg. Forty-five children presented non-detectable levels of testosterone. Thus, due to the small sample of testosterone and progesterone, these markers were not considered for further analysis.

### Measures of early-life adversity

#### Socioeconomic disadvantage (SES-D)

We developed a general index of socioeconomic disadvantage in Phase-I by including several indicators into a unidimensional factorial model. Variables retained were those with significant factor loadings (λ) in the discovery sample such as caregiver’s education (measured on a 5-point scale; 0 = some high school or less to 4 = Master’s degree or higher, *λ* = −0.86); family income (measured on a 5-point scale varying from 0 = less than $15,000 per year to 4 = more than $90,001 per year, *λ* = −0.91); caregiver’s perception on how safe their neighborhood is after dark (varying from 0 = completely safe to 4 = extremely dangerous, *λ* = 0.38), maternal job status (unemployed = 0, employed = 1, *λ* = −0.41), loss of employment by a member of the family other than the mother (no = 0, yes = 1, *λ* = 0.58), parents recent separation or divorce (no = 0, yes = 1, *λ* = 0.43). The Cronbach’s alpha for the SES-D was 0.74 and the factorial fit indices were adequate (*χ*^2^ = 16.08, df = 9, *p* = 0.053, Comparative Fit Index = 0.95, Tucker Lewis index = 0.91, root mean square error of approximation = 0.07). Other variables that were tested but did not fit well (non-significant *λ*s or worsened the fit indices) in the model were: if the caregiver was threatened or harassed and how often (12.50% less than once a year), family violence or abuse towards the caregiver or someone close (0.58% in the past 12 months), life-threatening illness or accidental injury of the caregiver or someone close (3.50% in the past 12 months), suspension of government funds (1.17% in the past 12 months), numbers of different jobs since pregnancy (*M* = 0.87, SD = 0.84), participation in a clinic or community-based service programs (88.02% never or less than once per week).

#### Caregiver depression

The short version of the Center for Epidemiological Studies Depression Scale^36^ (CESD) was administered to assess symptoms associated with depression in the caregivers (CESD-8).^37^ The CESD-8 is designed to assess depressive symptoms in the general population, more specifically the affective domain (depressed mood). The CESD-8 contains eight items assessing different symptoms that are indicators of depressed affect, positive affect, somatic and reduced activity, and interpersonal aspects.

#### Caregiver anxiety

We used the Generalized Anxiety Disorder scale (GAD-2^38^) to assess the levels of general anxiety in the caregivers. The GAD-2 scale consists of a self-report questionnaire that is the initial step for screening generalized anxiety disorder. It includes two items (*feeling nervous*, *anxious*, *or on edge*; and *not being able to stop or control worrying*) in which participants should rate how difficult it was for them to deal with each symptom (from 0 = *not at all* to 3 = *nearly every day*). Such items are critical components of anxiety disorders and the scale presented good sensitivity and specificity for the diagnosis of common anxiety disorders found in primary care.^38^

#### Family dysfunction

The Short General Functioning subscale of the McMaster Family Assessment Device (FAD^39^) was used to assess the family dynamics of the dyads. The FAD is grounded by the McMaster’s Model of Family Functioning.^40^ This framework assumes that family actors must be understood in integration with the rest of the family system, family structure and transactional patterns are important factors that influence the behaviors of family members. The FAD is comprised of six items scored on a 4-point scale (from 1 for strongly agree to 4 for strongly disagree), that assesses perceived family functioning, with higher scores indicating worse levels of family functioning.

### Data analysis procedures

Biochemical measures were summarized (e.g., mean, median, standard deviation, and interquartile range) and their distributions investigated. Cytokines and steroids values were positively skewed and were log_10_ transformed. Exploratory data analysis was performed using descriptive statistics, Pearson’s correlations, or analysis of variance for all variables used in the current study. Biomarkers log_10_ values were correlated with child age and sex yielding significant associations (*r*s ranged from −0.55 to 0.45 for age, and Cohen’s *d* effect size varied from −0.48 to −0.10 for sex), and thus we adjusted for these variables with linear regression analysis, and residuals were used in all further analyses. Body mass index (BMI) was not significantly associated with the biomarkers. Ethnicity was only significantly associated with cortisol levels, *F* (2, 73) = 4.90, *p* = 0.010, although it was not significant when included as a covariate in the regression with the early-life adversity predictors. Study site differences were also investigated and indicated differences in TNF-α in Phase-I (*F* [3,60] = 4.16, *p* = 0.010), and DHEA in Phase-II (*F* [2,51] = 14.12, *p* < 0.001, see Supplementary Table S2), that were considered a confounder for study site differences in income (*F* [3,60] = 66.03, *p* < 0.001, and *F* [2,51] = 41.03, *p* < 0.001, respectively). We included study site as a covariate for the TNF-α, but not for DHEA since study site was also not significant when included as a covariate with the early-life adversity predictors.

One regression was fitted for each children’s biochemical measure in which the socioeconomic disadvantage index, caregiver’s depression and anxiety, and family functioning served as predictors. Considering previous findings^41–43^ we expected to observe positive associations between the early-life adversity predictors and the stress response biomarkers, thus we considered one-sided significance levels for regression parameters and further adjusted for multiple comparisons using false discovery rate (FDR) correction. Adjusted significance levels were set at FDR *q* < 0.05. To ensure reliability and robustness of regression parameters, we undertook further analyses to sample composition using 500 bootstrapping with replacement and random resampling. Bootstrapped samples sizes were equal to the total number of complete cases of each biomarker. Finally, since interaction effects were previously observed between contextual and direct adversities,^16^ we also investigated the interaction of SES-D and FAD using the caregiver’s CESD-8 and GAD-2 scores as covariates on biochemical measures. Simple slope analysis was carried out in case of a significant interaction. Data were analyzed using R.^44^ The computation of the SES-D index was performed using the *mirt* package,^45^ and simple slopes for significant interactions were estimated using the *reghelper* package.^46^

## Results

Table 2 shows descriptive statistics and correlations for all the measures considered in both phases of the study. In Phase-I, higher socioeconomic disadvantage was significantly associated with higher levels of IL-1β, TNF-α, and DHEA. Higher levels of family dysfunction were associated with higher levels of cortisol and cortisone. In general, cytokines were not significantly associated with steroids except for IL-1β and cortisone (*r* = −0.33, *p* = 0.011). There were no significant correlations between IL-6, and IL-8 with SES-D, CESD-8, GAD-2, and FAD. In Phase-II, socioeconomic disadvantage was strongly associated with DHEA, caregiver depression was positively associated with cortisone, and higher levels of family dysfunction were associated with higher levels of IL-8 and lower levels of cortisol.

**Table 2:** Medians, interquartile range, and Pearson correlation coefficients for all measures.

Variables		Descriptive statistics		Correlations										
		Phase-I MD (IQR)	Phase-II MD (IQR)	1	2	3	4	5	6	7	8	9	10	11
1	TNF-α pg/mL	1.1 (1.3)	1.2 (1.6)	–	0.69**	0.57**	0.80**	−0.33	−0.12	−0.10	0.24*	−0.10	−0.10	−0.14
2	IL-1β pg/mL	25.8 (45.9)	18.4 (36.7)	0.59**	–	0.57**	0.71**	−0.12	−0.34*	−0.20	0.17*	−0.08	−0.06	−0.01
3	IL-6 pg/mL	1.9 (2.6)	2 (3.6)	0.62**	0.48**	–	0.64**	−0.22	−0.17	0.06	0.08	0.08	0.06	−0.03
4	IL-8 pg/mL	191 (343.6)	201 (317.7)	0.81**	0.65**	0.68**	–	−0.13	−0.26*	−0.15	0.15	0.03	0.01	0.02
5	Cortisol pg/mg	22.5 (83.1)	32.3 (123.5)	0.00	−0.02	0.05	0.00	–	0.51**	0.19	0.08	0.10	−0.02	0.28*
6	Cortisone pg/mg	20.3 (35.4)	24.4 (25.3)	0.11	0.05	0.23	0.00	0.38*	–	0.30*	0.03	0.01	0.03	0.30*
7	DHEA	9.6 (10.7)	8.3 (21.5)	−0.06	0.12	0.01	0.04	0.12	0.16	–	0.24*	0.00	0.08	0.02
8	SES-D	0.2 (1.4)	0.1 (1.5)	0.01	0.09	−0.16	0.01	0.04	−0.01	0.63**	–	0.18*	0.14	0.18*
9	CESD-8	4.0 (4)	5 (4)	0.02	0.01	0.01	0.03	0.03	0.29*	−0.04	0.02	–	0.54**	0.32**
10	GAD-2	0 (2)	0 (1)*	0.11	0.03	0.01	0.08	−0.03	−0.01	−0.03	0.11	0.50**	–	0.29**
11	FAD	8.0 (2)	6 (2)**	0.10	0.13	0.10	0.17*	−0.30*	−0.03	−0.01	0.06	0.35**	0.19*	–

Note: Phase-I correlations are presented above the diagonal, and Phase-II correlations are depicted below the diagonal. Biomarkers levels were log10 transformed to address skew and were age and sex adjusted.

*SES-D* socioeconomic disadvantage index, *CESD-8* Center for Epidemiological Depression scale, *GAD-2* Generalized Anxiety Disorder scale, *FAD* Family Assessment Device.

*p < 0.05; **p < 0.001.

**Fig. 1:**
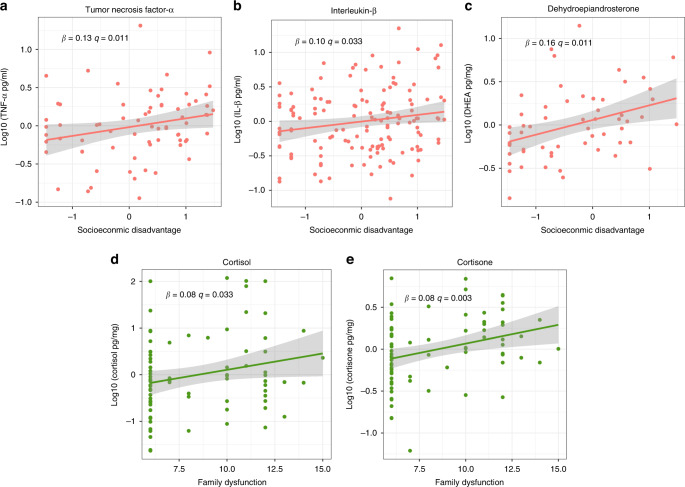
Statistically significant associations of early life adversities and biological responses in HERO Phase-I. Plots of significant linear regression lines for (**a**) tumor necrosis factor α, (**b**) interleukin 1β, and (**c**) dehydroepiandrosterone as a function of socioeconomic disadvantage, and cortisol (**d**) and cortisone (**e**) as a function of family dysfunction in Phase-I sample. Regression slopes are plotted for significant beta coefficients in each model, holding all other variables in the model at mean levels.

Phase-I multiple regression analyses showed that young children exposed to high levels of socioeconomic disadvantage had higher salivary levels of TNF-α, IL-1β, and hair DHEA after adjusting for family dysfunction, caregivers’ depression, and anxiety levels (Fig. 1a–c, respectively). Moreover, higher family dysfunction scores were found to be significantly associated with higher hair cortisol and cortisone levels in children, adjusting for all other factors (see Table 3 and Fig. 1d, e, respectively). These results were robust to sample composition using 500 bootstrapping with replacement and random resampling (see Supplementary Table S3). We also investigated if age and sex could influence the observed results through cross-product interactions. We did not observe significant interactions of socioeconomic disadvantage and family dysfunction with age and sex (see Supplementary Table S4).

**Table 3:** Phase-I regression coefficients of the association of early-life adversity measures with infants’ cytokines and steroids.

Outcome	SES-D			GAD-2			FAD			CESD-8			*N*	*R*^2^
	*B*	Std. *B*	FDR *q*	*B*	Std. *B*	FDR *q*	*B*	Std. *B*	FDR *q*	*B*	Std. *B*	FDR *q*		
TNF-α pg/mL	**0.13**^a^	**0.28**	**0.011**	0.01	0.14	0.362	−0.01	0.00	0.657	0.00	−0.28	0.407	71	0.10
IL-1β pg/mL	**0.10**^a^	**0.19**	**0.033**	−0.02	−0.05	0.362	0.00	−0.02	0.863	0.00	−0.04	0.407	142	0.04
IL-6 pg/mL	0.03	0.06	0.524	0.02	0.06	0.362	−0.02	−0.10	0.303	0.01	0.06	0.407	138	0.02
IL-8 pg/mL	0.07	0.14	0.122	−0.01	−0.02	0.438	−0.01	−0.04	0.702	0.01	0.05	0.407	139	0.02
Cortisol pg/mg	−0.02	−0.02	0.871	−0.08	−0.15	0.362	**0.08**^a^	**0.27**	**0.033**	0.03	0.13	0.407	79	0.09
Cortisone pg/mg	0.00	0.00	0.995	0.02	0.08	0.362	**0.05**^a^	**0.37**	**0.003**	−0.02	−0.21	0.319	79	0.10
DHEA pg/mg	**0.16**^a^	**0.35**	**0.011**	0.03	0.14	0.362	0.00	0.00	0.982	−0.03	−0.28	0.294	76	0.09

Note: bold values indicate FDR-adjusted significant associations (*q* < 0.05).

*SES-D* socioeconomic disadvantage index, *CESD-8* Center for Epidemiological Depression scale, *GAD-2* Generalized Anxiety Disorder scale, *FAD* Family Assessment Device, *Std.* standardized.

^a^Significant at 5% bootstrap levels.

**Fig. 2:**
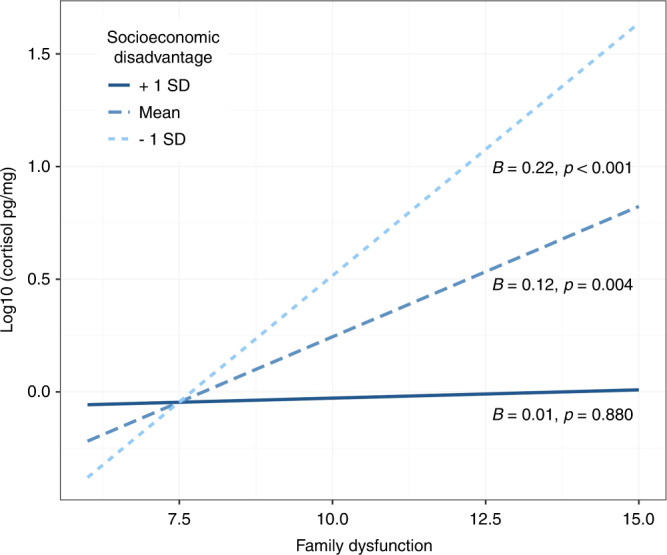
Socioeconomic disadvantage index moderates the association between family dysfunction and hair cortisol levels in young children in Phase-I. Linear regression analyses followed by simple slope analysis showed that children from low (-1 SD) and average levels of socioeconomic disadvantage had a significant positive association between hair cortisol and family dysfunction, whereas children exposed to high levels of socioeconomic vulnerability (+1 SD) showed no significant association between family dysfunction and hair cortisol levels.

A significant interaction effect between socioeconomic disadvantage and family dysfunction was observed only on hair cortisol levels (*B* = −0.13, *p* = 0.028). The effect observed for family dysfunction on cortisol levels changed as a function of socioeconomic disadvantage (see Fig. 2 for simple slopes). Children exposed to lower and average levels of socioeconomic disadvantage showed a significant positive association between hair cortisol and family dysfunction, whereas children exposed to high levels of socioeconomic vulnerability showed no significant association between family dysfunction and hair cortisol levels. This interaction effect was not significant for other biological markers (see Supplementary Table S4).

**Fig. 3:**
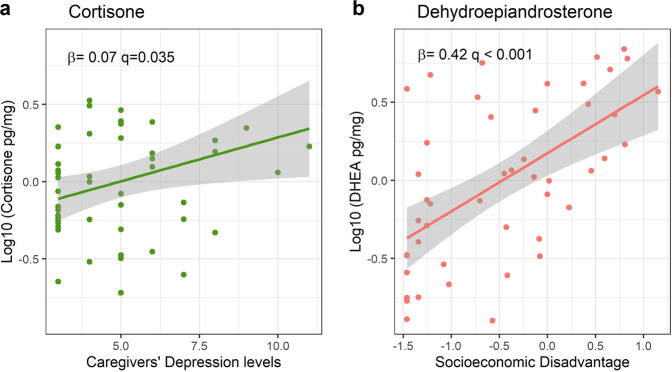
Statistically significant associations of early life adversities and biological responses in HERO Phase-II. Plots of significant linear regression lines for (**a**) cortisone as a function of caregivers‘ depression level, and (**b**) dehydroepiandrosterone as a function of socioeconomic disadvantage in Phase-II. Regression slopes are plotted for significant beta coefficients in each model, holding all other variables in the model at mean levels.

Replication analysis of TNF-α, IL-1β, cortisol, cortisone, and DHEA associations with socioeconomic disadvantage, caregivers’ depression, and anxiety levels in the Phase-II sample is depicted in Table 4. Higher levels of socioeconomic disadvantage were also associated with higher levels of DHEA (*B* = 0.42, *p* < 0.001, Bootstrap CI = 0.23: 0.55, Fig. 3a). Caregiver’s depression levels were positively associated with higher hair cortisone in infants but this association was not significant according to the bootstrapping procedure (*B* = 0.07, *p* = 0.035, Bootstrap CI = −0.01: 0.14, Fig. 3b). The observed significant negative association between family dysfunction and cortisol was not significant anymore when accounting for socioeconomic disadvantage, caregivers’ depression, and anxiety levels. We did not observe significant interactions of socioeconomic disadvantage and caregiver’s depression levels with age and sex (see Supplementary Table S4).

**Table 4:** Phase-II regression coefficients of the association of early-life adversity measures with infants’ cytokines and steroids.

Outcome	SES-D			GAD-2			FAD			CESD-8			*N*	*R*^2^
	*B*	Std. *B*	FDR *q*	*B*	Std. *B*	FDR *q*	*B*	Std. *B*	FDR *q*	*B*	Std. *B*	FDR *q*		
TNF-α	0.00	−0.01	0.935	0.08	0.19	0.216	0.01	0.04	0.352	−0.01	−0.10	0.320	126	0.03
IL-1β	0.06	0.11	0.247	0.05	0.09	0.334	0.01	0.05	0.352	−0.01	−0.05	0.420	126	0.02
Cortisol	0.00	0.00	0.980	0.05	0.05	0.379	−0.07	−0.25	0.352	0.01	0.03	0.421	49	0.05
Cortisone	−0.04	−0.08	0.562	−0.05	−0.09	0.334	−0.01	−0.09	0.352	**0.07**	**0.39**	**0.035**	50	0.13
DHEA	**0.42**^a^	**0.65**	**<0.001**	0.07	0.11	0.334	−0.01	−0.06	0.352	−0.03	−0.12	0.320	47	0.40

Note: bold values indicate FDR-adjusted significant associations (*q* < 0.05).

*SES-D* socioeconomic disadvantage index, *CESD-8* Center for Epidemiological Depression scale, *GAD-2* Generalized Anxiety Disorder scale, *FAD* Family Assessment Device, *Std.* standardized.

^a^Significant at 5% bootstrap levels.

## Discussion

Socioeconomic gradients in health are well documented across societies. The association between early-life adversity and greater risk for physical and mental health impairments in adulthood is similarly well supported by existing data. Although the evidence for these persistent disparities is irrefutable, their underlying pathophysiology remains unclear. Moreover, the frontiers of twenty-first-century biology indicate that these robust, population-level correlations obscure the compelling evidence of differential sensitivity to context, which makes individual-level predictions highly problematic. The magnitude of this challenge is underscored by recent analyses of prospectively collected data on adverse childhood experiences (ACEs) from two large birth cohorts that found that screening for ACE scores predicted mean group differences in both physical and mental health problems but was barely better than chance in predicting individual risk.^47^

A wide range of frameworks (both complementary and mutually exclusive) have been proposed to describe the underlying processes of the biological embedding of adversity. Some have proposed common pathways independent of the source or type of adverse experience or exposure.^46^ Others have presented evidence of differential effects on brain development based on responses to threat compared to deprivation.^9^ In this work, the understanding of early-life adversities and biological responses was framed as a developmental process, grounded by the multilevel biopsychosocial dynamic systems (e.g., developmental systems theory,^47^ the biological model of human development,^48^ and Sameroff’s 2010 unified theory of development^49^). More specifically the microsystem (where proximal processes occur)—classically defined as a pattern of activities, social roles, and interpersonal relations experienced by the developing person in a given face-to-face setting in the immediate environment^48^—was represented by measures concerning caregivers (e.g., mental health in the form of depression and anxiety), and family functioning. While distal adversities—which encompasses conditions that more indirectly impact the child^9^—were represented by the SES-D index.

Advances in the biology of adversity and resilience are beginning to unpack the heterogeneity in outcomes by investigating complex interactions among genetic factors, family and community environments, and developmental timing.^4^ These diverse perspectives present a rich agenda for deeper investigation. The implications for preventive intervention in the early childhood period are potentially transformative. In both cases, significant progress will require augmented capacity to measure key indicators of early biological embedding of adversity in real time.^12,50^

This study presents data from two phases of the feasibility study from The JPB Research Network on Toxic Stress. The HERO project was created to develop validated measures of stress activation and resilience in young children which could be incorporated into pediatric primary care practice. The findings from the initial feasibility study (Phase-I) show that different sources of early adversity (distal or and proximal) were associated with different biological responses (inflammation and HPA axis activation). Distal adversities—represented by a cumulative measure of socioeconomic indicators (e.g., caregiver education, family income, caregiver perception of neighborhood safety after dark, maternal job status, loss of employment from a member of the family other than the mother, and parents recent separation or divorce)—were associated with elevated levels of two potent pro-inflammatory cytokines (IL-1β and TNF-α) in saliva,^51,52^ and elevated levels of an adrenocortical product from the HPA axis (DHEA) in hair.^53^ One form of proximal adversity, family dysfunction, predicted levels of cortisone and cortisol in children’s hair samples, two active products of the adrenal cortex.^28^ We also observed a significant interaction effect between the socioeconomic disadvantage index and family dysfunction in predicting children’s hair cortisol levels. We did not find evidence of contextual or proximal adversity effects on children’s levels of IL-6 or IL-8.

In Phase-II, the SES-D measure was also positively associated with hair DHEA, and a form of proximal adversity (caregiver’s depression levels) was positively associated with cortisone levels. We note that the replication of Phase-I findings in Phase-II deserves caution as samples differed in terms of age and socioeconomic composition, since timing and duration of environmental disruptions play an important role in determining outcomes.^1^ Nonetheless, we believe that our replication efforts are a strength of our study, in times of replicability crises.^54,55^

Environmental influences on children’s physical health and development, and in particular on the HPA axis, have been well documented in the literature. Most of the risk factors studied have involved proximal adversities, such as child abuse or neglect,^56,57^ family instability and maternal unresponsiveness,^58^ and poor attachment.^59^ Studies that have explored the distinctive impact of broader contextual adversities (e.g., poverty) on HPA axis functioning in young children have yielded mixed results.^9^ For example, Vliegenthart et al.^60^ found that a neighborhood-level socioeconomic status score was significantly associated with hair cortisol and cortisone, while Ursache et al.^61^ only observed an association for parent education but not for family income. It is important to note that these studies did not simultaneously consider proximal adversities, including parent mental health or family function, as covariates in their statistical models.

In terms of pro-inflammatory markers, this study suggests that distal adversity had a stronger association in comparison with direct adversities. These results are consistent with a recent meta-analysis of data from 13 studies, 4 of which involved contextual adversities with significant associations and 9 of which examined proximal adversities.^3^ Most of the significant associations, reported in five studies, involved extreme forms of direct adversity such as sexual abuse, maltreatment, other childhood trauma, and living in a war zone. Results of studies that investigated relatively less intense forms of proximal adversity, like those considered in this work, were mixed (two out of four studies reported significant associations). Given that the reviewed studies were not entirely comparable, studies that investigated both distal and proximal adversities, and their independent effects on children’s inflammatory biomarker levels, are still scarce. Although our findings present preliminary data on a relatively small sample, they point to a greater susceptibility to inflammation in children exposed to an environment with greater socioeconomic disadvantage in the absence of extreme forms of proximal adversity, like observed in this community sample (only 0.58% reported extreme forms of adversity such as family violence).

Data on DHEA are even more scarce in the existing literature. Exposure to poverty was found to be associated with HPA axis reactivity (a composite obtained by dividing cortisol by DHEA-sulfate) in an adolescent sample.^62^ Another study of children exposed to maltreatment observed an atypical pattern of DHEA elevations only if maltreated children exhibited a resilient behavior phenotype.^53^ The associations of contextual adversity with IL-1β, TNF-α, and DHEA could reflect an attempt at DHEA adaptation to counter the apoptotic activities of pro-inflammatory cytokines.^19^ Since DHEA has anti-inflammatory and immunomodulatory effects, these specific results might be portraying an interrelated DHEA/cytokine biological response to socioeconomic disadvantage.

Correlations between socioeconomic factors and both family functioning and caregiver mental health have been documented extensively. For example, the hardships of poverty can undermine parents’ capacity to provide a health-promoting family environment.^58^ Consistent with previous research by Johnson et al.,^16^ where income interacted with attachment style, our investigation of the moderation of SES-D on FAD yielded a significant interactive effect in the prediction of hair cortisol levels in children. This finding indicated that family dysfunction was positively associated with hair cortisol for children with lower or average socioeconomic adversity, which is consistent with the literature examining salivary cortisol in children around this age.^62^ However, the interaction also showed that, for children exposed to an environment with high socioeconomic disadvantage, family dysfunction was not associated with cortisol levels. Although the non-significant association for those high in SES-D may seem contradictory at first, this result might be related to chronic activation of the HPA axis which often leads to a blunted cortisol response to stressful stimuli as reflected in normative down-regulation of the HPA axis following a sustained period of hyper-responsiveness,^63^ which is consistent with the allostatic load model.^64^ Since the socioeconomic disadvantage index moderator can be considered a stable measure, and hair cortisol levels reflect a measure of chronic HPA axis function,^65^ the interaction results might be capturing a subsample of participants with hypocortisolemia.^18,63^ One way to test this hypothesis is by investigating the diurnal variation of salivary cortisol, as hypocortisolism is related to low cortisol reactivity to an eliciting stimulus.^29^ For example, high hair cortisol combined with a flattened salivary cortisol slope was associated with more environmental risks.^65^ Without a dynamic measure of cortisol that assesses diurnal variation, preferably in the home environment, it was not possible to test this hypothesis. This interaction between distal and proximal adversities reveals the importance of considering dynamic and chronic biomarkers in future studies on pathophysiological mechanisms of early-life adversities.

The results of the present study illustrate the untapped potential of continuing advances in the biology of adversity and resilience and new measurement capacity for informing more effective preventive health care for young children facing adversity. Although the American Academy of Pediatrics recommends that primary care providers screen children for exposure to adverse experiences and provide recommendations for indicated services,^11^ there is a compelling need for new measures that capture individual variation in sensitivity to context to identify candidates for targeted, well-matched services and establish baseline indicators for assessing differential intervention effects. With these goals in mind, a better understanding of how the immune and HPA axis systems respond differently to distinct risk factors in early development could help identify physiological disruptions before overt symptoms appear. Such information could then guide the provision of tailored management approaches to reduce later disparities in stress-related diseases and disabilities.

There are a few limitations of the current study that should be highlighted. While hair steroids are expected to reflect a temporal average of HPA axis activation, salivary cytokines have higher fluctuations due to infections, neurodevelopmental conditions, and lifestyle influences.^3^ Although children screened for premature birth, congenital disorders, previous recent vaccination, and steroid medication use were excluded from the study sample, unreported infections may be a confounder. Another aspect to be considered is that although our samples were composed of a variety of adversities and markers of both endocrine and immune function with replication and bootstrapping for robustness efforts in a difficult to access developmental stage, our sample sizes were relatively small, and generalizability across ethnic groups, adversities interactions, and sex requires larger and more diverse samples. A third limitation is that we could not control for the intertwined, bidirectional relation between the HPA axis and the immune system,^18^ as HPA activity mediates and is mediated by immune system activity. For example, pro-inflammatory cytokines IL-1β and IL-6 stimulate corticotropin-releasing hormone, which triggers a cascade of responses leading to an elevation in cortisol release from the adrenal cortex. Cortisol, in turn, inhibits the production of pro-inflammatory cytokines.^18,28^ The cross-sectional design of the study is also a limitation for mediation analyses, and the lack of experimental manipulation precludes the determination of antecedents and consequents of the HPA and immune systems bidirectional interactions.

Despite these limitations, the results of this study indicate that two important biological response systems may react differently to diverse sources of adversity. These preliminary findings highlight the critical need for deeper investigation into the biological embedding of hardship and trauma in the early childhood years to advance our understanding of the pathophysiology that link identified sources of adversity to a wide range of stress-related, chronic conditions well into the adult years. To our knowledge, this is the first study that investigated different forms of early adversity and their distinctive biological signatures on the immune system and HPA axis in children under 5 years of age.

## Supplementary information

Supplementary Materials

## Data Availability

The data that support the findings of this study are available on reasonable request from the corresponding author, P.P.S. The data are not publicly available due to information that could compromise the privacy of research participants.

## References

[1] Nelson CA, Gabard-Durnam LJ (2020). Early adversity and critical periods: neurodevelopmental consequences of violating the expectable environment. Trends Neurosci..

[2] McLaughlin KA, Sheridan MA (2016). Beyond cumulative risk: a dimensional approach to childhood adversity. Curr. Dir. Psychol. Sci..

[3] Kuhlman KR, Horn SR, Chiang JJ, Bower JE (2019). Early life adversity exposure and circulating markers of inflammation in children and adolescents: a systematic review and meta-analysis. Brain Behav. Immun..

[4] Boyce WT, Levitt P, Martinez FD, McEwen BS, Shonkoff JP (2021). Genes, environments, and time: the biology of adversity and resilience. Pediatrics.

[5] Kessler RC (2010). Childhood adversities and adult psychopathology in the WHO world mental health surveys. Br. J. Psychiatry.

[6] Korkeila J (2010). Childhood adversities as predictors of incident coronary heart disease and cerebrovascular disease. Heart.

[7] Johnson J (2020). The extent to which childhood adversity and recent stress influence all-cause mortality risk in older adults. Psychoneuroendocrinology.

[8] Bhan N, Glymour MM, Kawachi I, Subramanian SV (2014). Childhood adversity and asthma prevalence: evidence from 10 US states (2009–2011). BMJ Open. Respir. Res..

[9] Wesarg C (2020). Identifying pathways from early adversity to psychopathology: a review on dysregulated HPA axis functioning and impaired self-regulation in early childhood. Eur. J. Dev. Psychol..

[10] Duffy KA, McLaughlin KA, Green PA (2018). Early life adversity and health-risk behaviors: proposed psychological and neural mechanisms. Ann. NY Acad. Sci..

[11] Kuhlman KR, Robles TF, Bower JE, Carroll JE (2018). Screening for childhood adversity: the what and when of identifying individuals at risk for lifespan health disparities. J. Behav. Med..

[12] Shonkoff JP, Boyce WT, Levitt P, Martinez FD, McEwen B (2021). Leveraging the biology of adversity and resilience to transform pediatric practice. Pediatrics.

[13] Gunnar, M. R. in *The Effects of Early Life Adversity on Neurobehavioral Development: Minnesota Symposium on Child Psychology* (ed. Nelson, C. A.). 163–200 (Psychology Press, 2000).

[14] Sheridan MA, McLaughlin KA (2014). Dimensions of early experience and neural development: deprivation and threat. Trends Cogn. Sci..

[15] Kuhlman KR, Chiang JJ, Horn S, Bower JE (2017). Developmental psychoneuroendocrine and psychoneuroimmune pathways from childhood adversity to disease. Neurosci. Biobehav. Rev..

[16] Johnson AB, Mliner SB, Depasquale CE, Troy M, Gunnar MR (2018). Attachment security buffers the HPA axis of toddlers growing up in poverty or near poverty: assessment during pediatric well-child exams with inoculations. Psychoneuroendocrinology.

[17] Kudielka BM, Buske-Kirschbaum A, Hellhammer DH, Kirschbaum C (2004). HPA axis responses to laboratory psychosocial stress in healthy elderly adults, younger adults, and children: impact of age and gender. Psychoneuroendocrinology.

[18] Reilly EB, Gunnar MR (2019). Neglect, HPA axis reactivity, and development. Int. J. Dev. Neurosci..

[19] Maninger N, Wolkowitz OM, Reus VI, Epel ES, Mellon SH (2009). Neurobiological and neuropsychiatric effects of dehydroepiandrosterone (DHEA) and DHEA sulfate (DHEAS). Front. Neuroendocrinol..

[20] Schury K (2017). Alterations of hair cortisol and dehydroepiandrosterone in mother-infant-dyads with maternal childhood maltreatment. BMC Psychiatry.

[21] Oitzl MS, Champagne DL, van der Veen R, de Kloet ER (2010). Brain development under stress: hypotheses of glucocorticoid actions revisited. Neurosci. Biobehav. Rev..

[22] Gunnar MR, Vazquez DM (2001). Low cortisol and a flattening of expected daytime rhythm: potential indices of risk in human development. Dev. Psychopathol..

[23] Koss KJ, Hostinar CE, Donzella B, Gunnar MR (2014). Social deprivation and the HPA axis in early development. Psychoneuroendocrinology.

[24] McLaughlin KA (2015). Causal effects of the early caregiving environment on development of stress response systems in children. Proc. Natl Acad. Sci. USA.

[25] Miller AH, Raison CL (2016). The role of inflammation in depression: from evolutionary imperative to modern treatment target. Nat. Rev. Immunol..

[26] Voltas N (2017). Are there early inflammatory biomarkers that affect neurodevelopment in infancy?. J. Neuroimmunol..

[27] Jiang NM (2017). Early life inflammation and neurodevelopmental outcome in Bangladeshi infants growing up in adversity. Am. J. Trop. Med. Hyg..

[28] Chrousos GP (1995). The hypothalamic–pituitary–adrenal axis and immune-mediated inflammation. N. Engl. J. Med..

[29] Koss KJ, Gunnar MR (2018). Annual Research Review: Early adversity, the hypothalamic–pituitary–adrenocortical axis, and child psychopathology. J. Child Psychol. Psychiatry.

[30] Carlo WA (2011). Cytokines and neurodevelopmental outcomes in extremely low birth weight infants. J. Pediatr..

[31] Businaro R (2016). Interleukin-18 modulation in autism spectrum disorders. J. Neuroinflammation.

[32] Measelle JR, Ablow JC (2018). Contributions of early adversity to pro-inflammatory phenotype in infancy: the buffer provided by attachment security. Attach. Hum. Dev..

[33] Tyrka AR, Parade SH, Valentine TR, Eslinger NM, Seifer R (2015). Adversity in preschool-aged children: effects on salivary interleukin-1β. Dev. Psychopathol..

[34] McKay HS (2017). Multiplex assay reliability and long-term intra-individual variation of serologic inflammatory biomarkers. Cytokine.

[35] Gao W (2013). Quantitative analysis of steroid hormones in human hair using a column-switching LC-APCI-MS/MS assay. J. Chromatogr. B Anal. Technol. Biomed. Life Sci..

[36] Radolf LS (1977). The CES-D Scale: a self report depression scale for research in the general population. Appl. Psychol. Meas..

[37] Briggs R, Carey D, O’Halloran AM, Kenny RA, Kennelly SP (2018). Validation of the 8-item Centre for Epidemiological Studies Depression Scale in a cohort of community-dwelling older people: data from The Irish Longitudinal Study on Ageing (TILDA). Eur. Geriatr. Med..

[38] Sapra A, Bhandari P, Sharma S, Chanpura T, Lopp L (2020). Using Generalized Anxiety Disorder-2 (GAD-2) and GAD-7 in a primary care setting. Cureus.

[39] de Haan KLB, Hafekost J, Lawrence D, Sawyer MG, Zubrick SR (2015). Reliability and validity of a short version of the General Functioning Subscale of the McMaster Family Assessment Device. Fam. Process.

[40] Byles J, Byrne C, Boyle MH, Offord DR (1988). Ontario Child Health Study: reliability and validity of the General Functioning Subscale of the McMaster Family Assessment Device. Fam. Process.

[41] Gray NA (2018). Determinants of hair cortisol concentration in children: a systematic review. Psychoneuroendocrinology.

[42] Lacey RE (2020). Adverse childhood experiences and early life inflammation in the Avon longitudinal study of parents and children. Psychoneuroendocrinology.

[43] Baranov V (2022). Effects of a maternal psychosocial intervention on hair derived biomarkers of HPA axis function in mothers and children in rural Pakistan. SSM Ment. Health.

[44] R Core Team. R: A language and environment for statistical computing. https://www.r-project.org (2019).

[45] Chalmers RP (2012). mirt: A multidimensional item response theory package for the R environment. J. Stat. Softw..

[46] Hughes, J. reghelper: Helper functions for regression analysis. https://cran.r-project.org/package=reghelper (2020).

[47] Lerner, R. M. in *Handbook of Child Psychology: Theoretical Models of Human Development*, Vol. 1 (ed. Lerner, R. M.) 1–18 (John Wiley & Sons Inc., 2006).

[48] Bronfenbrenner, U. & Morris P. A. in *Handbook of Child Psychology: Theoretical Models of Human Development*, Vol. 1 (ed. Lerner, R. M.) 793–828 (John Wiley & Sons Inc., 2006).

[49] Sameroff A (2010). A unified theory of development: a dialectic integration of nature and nurture. Child Dev..

[50] Shonkoff JP (2022). Translating the biology of adversity and resilience into new measures for pediatric practice. Pediatrics.

[51] Lopez-Castejon G, Brough D (2011). Understanding the mechanism of IL-1β secretion. Cytokine Growth Factor Rev..

[52] Olmos G, Lladó J (2014). Tumor necrosis factor alpha: a link between neuroinflammation and excitotoxicity. Mediators Inflamm..

[53] Cicchetti D, Rogosch FA (2007). Personality, adrenal steroid hormones, and resilience in maltreated children: a multilevel perspective. Dev. Psychopathol..

[54] Moonesinghe R, Khoury MJ, Janssens ACJW (2007). Most published research findings are false—but a little replication goes a long way. PLoS Med..

[55] Gertler P, Galiani S, Romero M (2018). How to make replication the norm. Nature.

[56] Baldwin JR (2021). Population vs individual prediction of poor health from results of adverse childhood experiences screening. JAMA Pediatr..

[57] Smith KE, Pollak SD (2021). Rethinking concepts and categories for understanding the neurodevelopmental effects of childhood adversity. Perspect. Psychol. Sci..

[58] Suor JH, Sturge-Apple ML, Davies PT, Cicchetti D, Manning LG (2015). Tracing differential pathways of risk: associations among family adversity, cortisol, and cognitive functioning in childhood. Child Dev..

[59] Roque L, Veríssimo M, Oliveira TF, Oliveira RF (2012). Attachment security and HPA axis reactivity to positive and challenging emotional situations in child-mother dyads in naturalistic settings. Dev. Psychobiol..

[60] Vliegenthart J (2016). Socioeconomic status in children is associated with hair cortisol levels as a biological measure of chronic stress. Psychoneuroendocrinology.

[61] Ursache A, Merz EC, Melvin S, Meyer J, Noble KG (2017). Socioeconomic status, hair cortisol and internalizing symptoms in parents and children. Psychoneuroendocrinology.

[62] Bush NR, Obradović J, Adler N, Boyce WT (2011). Kindergarten stressors and cumulative adrenocortical activation: the “first straws” of allostatic load?. Dev. Psychopathol..

[63] Bunea IM, Szentágotai-Tǎtar A, Mil AC (2017). Early-life adversity and cortisol response to social stress: a meta-analysis. Transl. Psychiatry.

[64] Juster RP, McEwen BS, Lupien SJ (2010). Allostatic load biomarkers of chronic stress and impact on health and cognition. Neurosci. Biobehav. Rev..

[65] Flom M, John AM, Meyer JS, Tarullo AR (2017). Infant hair cortisol: associations with salivary cortisol and environmental context. Dev. Psychobiol..

