# Supplementary material for: Linking specific biological signatures to different childhood adversities: findings from the HERO project
Source: Pediatr Res. 2023 Jan 17;94(2):564–74. doi: 10.1038/s41390-022-02415-y (PMC10382309; doi:10.1038/s41390-022-02415-y)

Supplementary Online Content

Linking specific biological signatures to different forms of childhood adversities: Findings from the Health's Early Roots & Origins (HERO) project.

Table S1. Spearman's rank correlation between collected hair mass and cortisol, cortisone, and dehydroepiandrosterone (DHEA) concentrations.

Table S2. Biomarkers' concentrations by study's site.

Table S3. Bootstrapped regression coefficients of the association of early life adversity measures with infants' cytokines and steroids.

Table S4. Interaction cross-product regression coefficients of the association of early life adversity measures with infants' cytokines and steroids.

Table S.5. Regression coefficients with the interaction of Socioeconomic Disadvantage and Family Function in HERO Phase-I.

Graphical summary

Table S1. Spearman's rank correlation between collected hair mass and cortisol, cortisone, and dehydroepiandrosterone (DHEA) concentrations.

Biomarkers	Hair mass (*mg*)			
	Phase-I		Phase-II	
	rho	*p*	*rho*	*p*
Cortisol	-0.11	0.324	-0.23	0.079
Cortisone	-0.01	0.937	-0.11	0.407
DHEA	-0.13	0.273	-0.20	0.142

Table S2. Biomarkers' concentrations by study's site.

PHASE-I						
Biomarkers	Study site				Statistic	
	Site 1<br>N= 53<br>*M* (*SD*)	Site 2<br>N=10<br>*M* (*SD*)	Site 3<br>N=38<br>*M* (*SD*)	Site 4<br>N=59<br>*M* (*SD*)	*F*	*p*
TNF-α	0.07 (0.46)	-0.46 (0.63)	-0.18 (0.43)	0.16 (0.3)	4.16	0.010
IL-1β	-0.05 (0.45)	0.13 (0.26)	-0.02 (0.52)	0.09 (0.48)	0.87	0.459
IL-6	0.07 (0.49)	-0.22 (0.35)	-0.01 (0.42)	0.02 (0.51)	0.70	0.555
IL-8	-0.03 (0.46)	-0.21 (0.48)	0.03 (0.48)	0.08 (0.46)	0.94	0.422
Cortisol	-0.05 (0.93)	0.04 (0.14)	0.28 (1.08)	-0.14 (0.61)	0.68	0.569
Cortisone	0 (0.35)	0.06 (0.49)	0.11 (0.45)	-0.11 (0.5)	0.73	0.540
DHEA	-0.05 (0.43)	-0.23 (0.27)	0.09 (0.27)	0.06 (0.47)	0.90	0.448
PHASE-II						
Biomarkers	Study site				Statistic	
	Site 1<br>N= 50<br>*M* (*SD*)	Site 2<br>N= 9<br>*M* (*SD*)	Site 3<br>N= 43<br>*M* (*SD*)	Site 4<br>N= 43<br>*M* (*SD*)	*F*	*p*
TNF-α	0.08 (0.34)	-0.05 (0.63)	0.01 (0.38)	0.12 (0.42)	0.75	0.524
IL-1β	0.04 (0.51)	-0.17 (0.9)	0.04 (0.5)	0.11 (0.42)	0.73	0.539
IL-6	0.13 (0.48)	-0.01 (0.47)	-0.11 (0.53)	0.01 (0.41)	1.80	0.149
IL-8	0.05 (0.43)	0.04 (0.8)	0.13 (0.48)	0 (0.5)	0.52	0.668
Cortisol	-0.08 (0.84)	-	0.16 (0.78)	-0.05 (0.96)	0.41	0.664
Cortisone	-0.02 (0.44)	-	-0.02 (0.32)	-0.03 (0.24)	0.00	0.998
DHEA	-0.23 (0.47)	-	0.36 (0.33)	0.44 (0.35)	14.12	<0.001

Table S3. Bootstrapped regression coefficients of the association of early life adversity measures with infants' cytokines and steroids.

Outcome	SES-D		GAD-2		FAD		CES-D	
	B	Boot CI	B	Boot CI	B	Boot CI	β	Boot CI
TNF-α pg/ml	**0.13**	**[0.04 : 0.23]**	0.01	[-0.10 : 0.05]	-0.01	[-0.05 : 0.01]	0.00	[-0.03 : 0.02]
IL-1β pg/ml	**0.10**	**[0.01 : 0.20]**	-0.02	[-0.09 : 0.05]	0.00	[-0.03 : 0.02]	0.00	[-0.03 : 0.02]
IL-6 pg/ml	0.03	[-0.06 : 0.12]	0.02	[-0.09 : 0.11]	-0.02	[-0.05 : 0.01]	0.01	[-0.03 : 0.05]
IL-8 pg/ml	0.07	[-0.02 : 0.16]	-0.01	[-0.08 : 0.08]	-0.01	[-0.04 : 0.02]	0.01	[-0.02 : 0.03]
Cortisol pg/mg	-0.02	[-0.29 : 0.23]	-0.08	[-0.20 : 0.05]	**0.08**	**[0.01 : 0.16]**	0.03	[-0.04 : 0.10]
Cortisone pg/mg	0.00	[-0.13 : 0.12]	0.02	[-0.04 : 0.08]	**0.05**	**[0.02 : 0.09]**	-0.02	[-0.04 : 0.01]
DHEA pg/mg	**0.16**	**[0.03 : 0.27]**	0.03	[-0.04 : 0.10]	0.00	[-0.04 : 0.04]	-0.03	[-0.06 : 0.00]

Note. Bold lettering indicates 500 bootstrapping resampling significant associations. Boot CI = 2.5 and 97.5 percentiles. SES-D: Socioeconomic disadvantage index; CES-D: Center for Epidemiological Depression scale; GAD-2: Generalized Anxiety Disorder scale; FAD: Family Assessment Device.

Table S4. Interaction cross-product regression coefficients of the association of early life adversity measures with infants' cytokines and steroids.

Interactions by age

Outcome	*Phase-I*				*Phase-II*			
	SES-D		FAD		SES-D		CES-D	
	β	*FDR* q	B	*FDR* q	B	*FDR* q	β	*FDR* q
TNF-α pg/ml	0.00	0.893	0.00	0.295	0.00	0.914	0.00	0.698
IL-1β pg/ml	0.00	0.918	0.00	0.867	-0.01	0.448	0.00	0.460
IL-6 pg/ml	0.00	0.893	0.00	0.426	0.00	0.914	0.00	0.460
IL-8 pg/ml	0.00	0.893	0.00	0.481	-0.01	0.531	0.00	0.460
Cortisol pg/mg	0.02	0.135	-0.01	0.191	0.02	0.914	-0.01	0.698
Cortisone pg/mg	0.00	0.918	0.00	0.295	0.00	0.996	0.00	0.911
DHEA pg/mg	0.00	0.918	0.00	0.481	0.00	0.996	0.00	0.934

Interactions by sex

Outcome	*Phase-I*				*Phase-II*			
	SES-D		FAD		SES-D		CESD-8	
	B	*FDR* q	B	*FDR* q	B	*FDR* q	β	*FDR* q
TNF-α pg/ml	0.00	0.893	0.00	0.295	0.00	0.914	0.00	0.698
IL-1β pg/ml	0.00	0.918	0.00	0.867	-0.01	0.448	0.00	0.460
IL-6 pg/ml	0.00	0.893	0.00	0.426	0.00	0.914	0.00	0.460
IL-8 pg/ml	0.00	0.893	0.00	0.481	-0.01	0.531	0.00	0.460
Cortisol pg/mg	0.02	0.135	-0.01	0.191	0.02	0.914	-0.01	0.698
Cortisone pg/mg	0.00	0.918	0.00	0.295	0.00	0.996	0.00	0.911
DHEA pg/mg	0.00	0.918	0.00	0.481	0.00	0.996	0.00	0.934

Note. SES-D: Socioeconomic disadvantage index; CESD-8: Center for Epidemiological Depression scale; GAD-2: Generalized Anxiety Disorder scale; FAD: Family Assessment Device.

Table S.5. Regression coefficients with the interaction of Socioeconomic Disadvantage and Family Function in HERO Phase-I.

Outcome	SES		GAD-2		FAD		CESD-8		SES x FAD		R^2
	B	*p*	B	*p*	B	*p*	B	*p*	β	*FDR q*	
TNF-α pg/ml	0.22	0.248	-0.02	0.317	-0.01	0.430	0.00	0.720	-0.01	0.496	0.11
IL-1β pg/ml	0.09	0.578	0.00	0.856	0.00	0.878	0.00	0.582	0.00	0.496	0.04
IL-6 pg/ml	-0.10	0.559	0.00	0.950	-0.02	0.313	0.00	0.677	0.02	0.467	0.02
IL-8 pg/ml	0.07	0.671	0.00	0.795	0.00	0.805	0.00	0.659	0.00	0.496	0.02
Cortisol pg/mg	0.71	0.059	-0.04	0.274	0.04	0.260	0.03	0.176	**-0.13**	**0.028**	0.15
Cortisone pg/mg	-0.06	0.737	0.01	0.397	0.05	0.005	-0.01	0.216	0.01	0.496	0.12
DHEA pg/mg	-0.02	0.909	0.01	0.732	0.00	0.968	-0.01	0.321	0.02	0.467	0.09

Note. Bold lettering indicates FDR adjusted significant interaction ($p < 0.05$). SES-D: Socioeconomic disadvantage index; CESD-8: Center for Epidemiological Depression scale; GAD-2: Generalized Anxiety Disorder scale; FAD: Family Assessment Device.

Graphical Summary
HERO Phase I

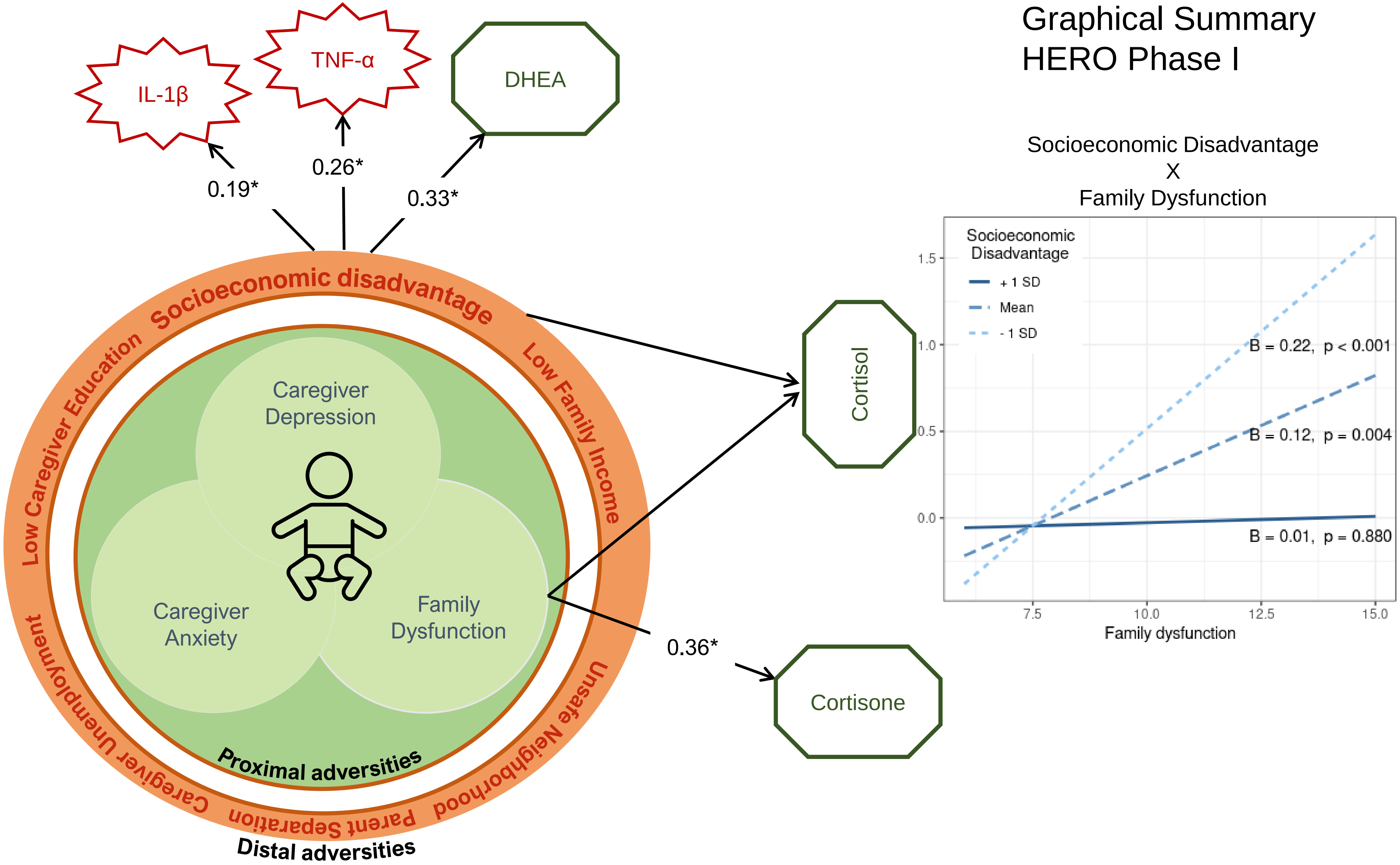

Supplement: Supplementary file 1 — Supplementary Materials [file 41390_2022_2415_MOESM1_ESM.pdf]
